# Screening and Isolation of Natural Antioxidants from *Acer Ginnala* Max by TLC-MS/MS Guided Bioautographic Method

**DOI:** 10.22037/ijpr.2019.1100690

**Published:** 2019

**Authors:** Jia-Ming Sun, Yinan Guo, Jie Zhang, Hui Zhang, Zhe Lin

**Affiliations:** a *Jilin Ginseng Academy, Changchun University of Chinese Medicine, Changchun 130117, China.*; b *The Affiliated Hospital of Changchun University of Traditional Medicine, Changchun University of Chinese Medicine, Changchun 130021, China.*; c *School of Pharmacy,* *Changchun University of Chinese Medicine, Changchun 130117, China.*

**Keywords:** Antioxidants, Acer ginnala Max., TLC, DPPH, ESI/MS

## Abstract

A rapid and simple method has been developed for the screening and identification of natural antioxidants from the leaves of *Acer* *ginnala *Maxim (AG). The process is that upon reaction with 1,1-diphenyl-2-picrylhydrazyl (DPPH), the white yellow spots of compounds with potential antioxidant effects will be significantly observed on the thin-layer chromatography (TLC), and possible structures will be presumed by the ESI/MS technique. Using the improved approach, 6 compounds in the AG extract were found to possess a potential antioxidant activity. They were speculated as quercetin-3-*O-α-L*-(3"-galloyl)-rhamnoside **(1)**, quercetin-3-*O-α-L*-(2"-galloyl)-rhamnoside **(2)**, quercetin-3-*O-α-L*-(2"-galloyl)-arabinopyranoside **(3)**, acertannin **(4)**, gallic acid **(5), **and methyl gallate **(6)**. In addition, we were still found that compounds** 2**, **3, **and **5 **had favorable antioxidant activity from the scannogram of the DPPH reaction plate. As a result, the isolated 6 compounds structures were in accordance with the presumed structures. Furthermore, the free radical scavenging capacities of the available identified compounds were also investigated. Compounds** 2**, **3,** and **5** showed significant DPPH^.^Scavenging capacities, with IC_50_ values of 2.83 μg/mL, 2.34 μg/mL, and 1.86μg/mL, respectively. The results indicated that this newly improved method could be widely applied for rapid screening and identification of natural antioxidants from Chinese herbal medicines.

## Introduction

Free radicals are considered to be important causative factors in the development of many physiological and pathological phenomena such as cancer, inflammation, and aging (1-3). Therefore, it is very important to keep the content of free radicals to a certain lower value. It is believed that antioxidant protects bodies from free radicals damages and thus plays a key role in the prevention of age-related diseases (4).

Natural antioxidants have many different types such as phenolic acids, flavonoids, and tannins, with more diversity in structure and bioactivity, and less toxicity (5). As a result, there has been an increasing interest in naturally occurring antioxidants in recent years. Availability of a simple and rapid method combining identification and screening of the potential antioxidants from Chinese herbal medicines is essential (6-8). However, the conventional approach to find bioactive components was with certain shortcomings like time-consuming, arduous, and less efficient for screening bioactive components from Chinese herbal medicines. Up to now, there would not be a useful approach for the identiﬁcation of major antioxidants in biological samples by use of the technique that combined TLC-DPPH test with the ESI/MS analysis.

AG is traditionally used as medicinal plant in China, Korea, and Japan for treating high blood pressure, coronary heart disease, and liver injury with special effects. Modern pharmacological studies showed that AG exhibited a broad spectrum of biological activities, such as antibacterial, anti-inflammatory, and hepato-protective effects (9-11). In addition, the recent studies indicated that AG also exhibited good antioxidant activities, which may partly be responsible for some of its medicinal functions (12). Han and Lu purified three antioxidant compounds, quercetin-3-O-α-L-rhamnopyranoside methyl gallate and aceritannin, from the methanol extract of AG (13, 14). Furthermore, Lu performed a components and radical scavenging activity analysis to Gao-Cha, which is a traditional Chinese health tea made from AG. In this tea they found another three antioxidant components, which were 3, 4, 5-trihydroxybenzoate, quercetin-3-O-α-rhamnopyranoside and 2, 6-bis (3, 4, 5-trihydroxybenzoyl)-aceritol, with four possible radical scavengers, such as ginnalin A and B, 2″-O-Galloylquercitrin and 3″-O-Galloyl-quercitrin. Based on our research early we think there are not only ten antioxidant compounds in AG. However, no strategy has been presented for rapid screening and identiﬁcation of natural antioxidants from AG.

The aim of this study was to develop a new rapid and simple method using TLC-DPPH-ESI/MS techniques to find more natural antioxidants in AG. Moreover, the free radical scavenging capacities of several identified compounds were confirmed on the basis of conventional spectrophotometric DPPH scavenging capacity assay.

## Experimental

*Plant materials*

The aerial part of *A. ginnala* was collected in Jilin Province, China, in August, 2010, and authenticated by Prof. Hui Zhang, Development Center of Traditional Chinese Medicine and Bioengineering, Changchun University of Chinese Medicine, Changchun, PR China. A voucher sample of the plant (20100825) was deposited in School of Traditional Chinese Materia Medica, Changchun University of Chinese Medicine.

*Instrumentation and reagents*

Thin-layer chromatography camera system (CAMAG, Switzerland). Thin-layer chromatography video scanning software was measured with wavelength 254nm, slit 6.0 mm x 0.45 mm and scanning speed of 20 mm·s^-1^(CAMAG, Switzerland). Electrospray ionization (ESI) mass spectra used to perform the studies was a 6320 ion trap LC/MS from Agilent. HPLC analysis was performed on an Agilent 1100 series HPLC (Agilent, USA) separations was performed on a C18 preparative column (Waters Sunfire C18 10.0 mm × 150 mm, 5μm) supplied by Waters, America. The methanol and water were used as the mobile phases A and B, respectively, the optimized HPLC elution procedures were conducted as follows: 0-25 min, 80%-90% A; 25-40 min, 90-90% A. The flow-rate was 0.3mL/min and the column temperature was maintained at 30℃. The chromatogram was recorded at 280 nm. Polyamide TLC plates purchased from Taizhou (Zhejiang, China) were used for TLC bioautography analysis. ^1^H and ^13^C NMR spectra were recorded on Bruker AM-400 spectrometer using DMSO-*d*_6_ as solvents.

1, 1-Diphenyl-2-picrylhydrazyl radical (DPPH) was purchased from Sigma (St.Louis, MO, USA). All solvents used for chromatography were of HPLC grade and obtained from Tedia (Fairfield, OH, USA). All other chemicals were of analytical grade without further purification. Reverse osmosis Milli-Q water (Millipore, Bedford, MA, USA) was used for the preparation of deionized water. 

*Isolation and Extraction*

The air-dried and powdered leaves of* A. ginnala* Max (2.2 kg) were extracted by maceration with MeOH for seven days at room temperature and the process was repeated twice. After ﬁltration, the combined MeOH extract was concentrated under reduced pressure at 45 °C to yield crude extract (500 g). The extract was suspended in distilled water and then extracted with PE, EtOAc, and n-BuOH successively. The EtOAc phase was concentrated to give a residue (80 g). The soluble extract was subjected to silica gel CC (100–200 mesh) eluted with a CHCl_3_–MeOH (10:0–0:10, v/v) gradient to afford five fractions (Fr.1–Fr.5) on the basis of TLC analysis. Fr.2 was further subjected to silica gel column and eluted with a CHCl_3_–MeOH (9:1–0:1, v/v) gradient to afford four subfractions (Fr.2A–Fr.2D). Subfraction 2A (2.7 g) was repeatedly subjected to a silica gel column (CHCl_3_–MeOH 8:2–6:4) puriﬁed by HPLC [MeOH/H_2_O: 45–55%, 2 mL/min, 254 nm, Sunfire prep. C18 column (Waters) (10×150 mm i.d., 5 mm)] to afford 1 (12 mg), 2 (12 mg), 3 (30 mg). Subfraction 2B (2.2 g) was passed over a Sephadex LH-20 column using CHCl_3_–MeOH (1:1) as the mobile phase to give 4 (35 mg). Subfraction 2C (40 mg) was shown the presence of one main compound signal by TLC analysis to give 5 (40 mg). A little portion of Subfraction 2D (1.6 g) was further puriﬁed by sephadex LH-20 and then RP-8 CC to give 6 (25 mg).

**Figure 1:**
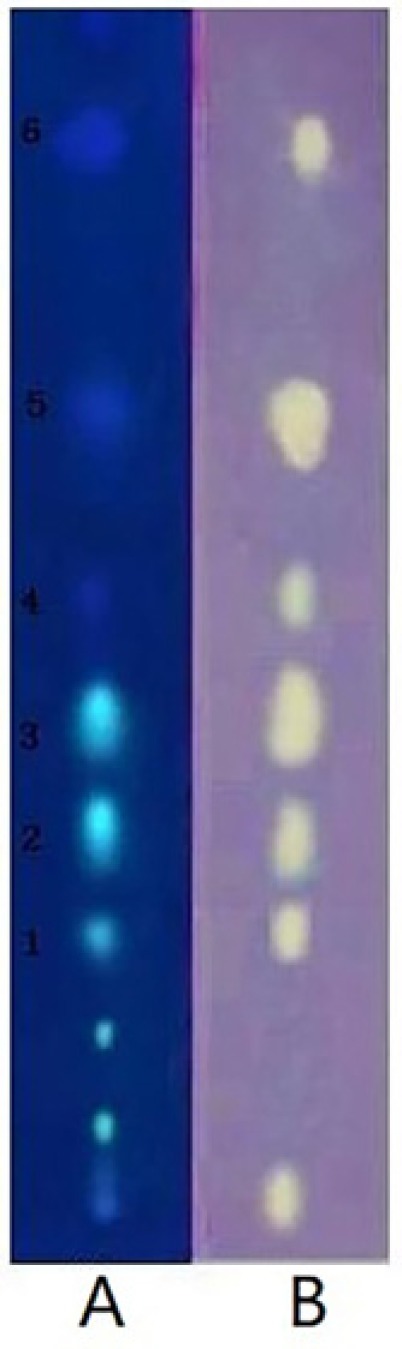
Polyamide TLC plates stained under UV 254nm (1A), with 0.4% DPPH. solution in methanol and visualized(1B) under visible light. AGE-80 was applied as dots on TLC layer. The spots marked with 1-6 indicate compounds with DPPH. scavenging activities

**Figure 2:**
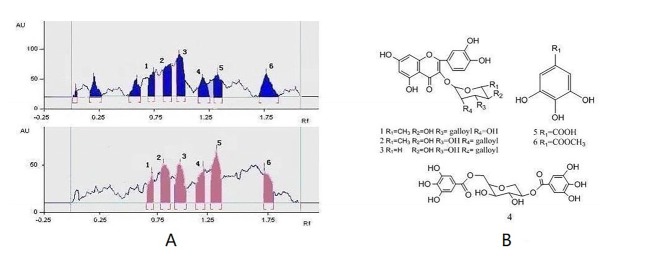
A: with the thin-layer chromatography video scanning software to scan the polyamide thin-layer plate 1A and 1B with wavelength 254nm, slit 6.0 mm*0.45 mm and speed of 20 mm.s-1; B: Chemical structures of compounds 1-6 isolated from leaves of *A. ginnala *Max

**Figure 3:**
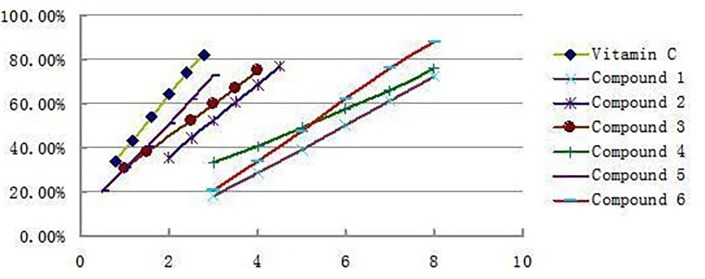
Dose effects of the pure isolates DPPH reactions. The absorbance at 517nm of the reactions was measured at minute 30 of the reaction. All the tests were conducted in duplicate, and the means were used

**Table 1:** 1H and 13C NMR data for compounds 1-6 at 400 MHz for protons and 100 MHz for carbons

**Carbon**	**1**	**2**	**3**	**4**	**5**	**6**	**1**	**2**	**3**	**4**
1				66.6	120.5	122.3				
2	156.4	156.4	156.3	71.5	108.7	110.3				
3	134.5	133.1	133.3	75.7	145.4	146.4				3.93 (dd, 2.2)
4	177.7	177.4	177.5	71.6	137.9	139.1				
5	161.2	161.2	161.2	83.8	145.4	146.4				3.56(d,11)
6	98.7	98.7	98.7	83.8	108.7	110.3	6.21(d,2.1)	6.20,s	6.21,s	3.54(d,5)
7	166.2	164.2	164.1							
8	93.6	93.6	93.6				6.41(d,2.1)	6.40,s	6.40,s	
9	157.3	157.2	157.4							
10	102.1	103.9	103.9							
1′	120.6	120.6	120.7	121.2						7.04(2H,s)
2′	112.8	115.5	115.6	109.6			7.30(d,6.4)	6.88(d,1.95)	7.30(d,2.05)	6.98(d,11.8)
3′	145.4	145.2	145.5	146.1						
4′	148.5	148.5	148.5	140.3						
5′	115.5	115.6	115.8	146.1			6.89(d,8)	6.94(d,8.31)	6.87(d,8.25)	
6′	121.3	121.1	121.1	109.6			7.31(d,6.4)	7.04(dd,8.31,1.95)	7.31(dd,8.25,2.05)	6.98(d,11.8)
7′				165.9						
1′′	98.3	98.3	98.4	121.0			5.45(d,4.8)	5.49(d,1.3)	5.45(d,1.4)	7.04(2H,s)
2′′	67.4	71.6	71.5	109.5			4.27(br,s)	5.46(dd,1.84)	5.44(dd,1.82)	6.94(d,11.8)
3′′	73.7	68.5	68.5	146.1			5.01(dd,6.4)			
4′′	68.4	71.6	71.5	140.3						
5′′	70.9	70.6	70.7	146.1						
6′′	17.5	17.5		109.5			0.88(d,4.8)	0.83(d,6.01)		6.94(d,11.8)
7′′				165.6						
1′′′	119.9	119.1	119.2							
2′′′	108.9	108.8	108.9				7.04,s	6.97,s	6.97,s	
3′′′	145.3	145.4	145.4							
4′′′	138.2	138.5	138.5							
5′′′	145.3	145.4	145.6							
6′′′	108.9	108.8	108.8				7.04,s	6.97,s	6.97,s	
7′′′	167.7	164.9	164.9							
C=O					167.4	167.7				

*DPPH radical scavenging*

DPPH radical scavenging assay was performed according to the method reported in with some modiﬁcations (15,16). Brieﬂy, an aliquot of 50 μL of different concentrations of each extract and individual pure compounds in methanol was added to 50 μL of 0.3mM DPPH methanol solution. After gentle mixing and 30 min of standing at room temperature, absorbance at 517 nm was measured using a spectrophotometer. All measurements were performed in triplicate and the result was an average of three determinations. The DPPH RSA (%) was calculated by the following equation: Radical scavenging activity (%) = [(A_0_-A_1_/A_0_)]*100% (where A_0_ was the absorbance of the reagent blank, and A_1_ was the absorbance with antioxidants). In addition, the IC_50_ value of each sample was obtained by plotting the percent DPPH scavenging of each concentration of an antioxidant sample against the sample concentrations. Vitamin C was used as a positive control. 

*TLC bioautography analysis of Fr.2*

DPPH (0.4g) was dissolved in 100 mL MeOH during the bioassay. An aliquot of Fr.2 methanol solution (1 mg/mL, 2mL) was directly deposited as spots onto the two polyamide TLC plates. Polyamide TLC plates were developed in a presaturated solvent chamber with methanol–water (1:0.4) as developing reagents until the solvent front reached 1 cm from the top of plates. The developed polyamide TLC plates were then removed from the chamber, and dried absolutely with a hair dryer (800 W). One plate was sprayed with 0.4% DPPH solution, then blown quickly with cold wind from a hair dryer until no free liquid flowing on it. The other TLC plate was monitored under UV light at 254nm. At the same time, the thin-layer chromatography video scanning software was used to scan the two polyamide thin-layer plates with wavelength 254nm, slit 6.0mmx0.45mm and scanning speed of 20 mm·s^-1^.

*Combination of TLC-ESI/MS analysis research on Fr.2*

Scraped the polyamide powder of the spots with the potential antioxidant activity into a vial, and added an aliquot of methanol, jolted and filtered with a 0.45 µm membrane filter. Then, the obtained solution was analyzed by ESI/MS. The analysis was carried out on Agilent 1100 HPLC/MSD Trap mass spectrometer (Agilent, Wilmington, Germany) equipped with an electrospray ionization source was used in positive ion mode using full scan mode and the mass range from 50 to 1200 m/z. The conditions of the ESI source were as follows: drying gas (N_2_) flow rate, 9.0 L/min; drying gas temperature, 350 ° ; nebulizer, 35 psi; capillary voltage, 4000 V; fragmentor 200 V; skimmer voltage, 60 V. Auto MS^2 ^mode of Mass spectrometer was chosen to analyze the sample. The data recorded was processed with the Applied HPLC-MSD ChemStation software system.

## Results and Discussion

*DPPH scavenging activity of extracts*

Using DPPH method to the AG antioxidant activity part carries on the analysis, calculated PE, CHCl_3_, EtOAc, n-BuOH, and H_2_O dry extracts with the IC_50 _value of 35.76 µg/mL, 12.55 µg/mL, 1.88 µg/mL, 3.44 µg/mL, and 31.25 µg/mL, respectively. Compared with vitamin C control group, EtOAc extract showed favourable antioxidant activity and the activity decreased as follows: EtOAc extract > n-BuOH extract > CHCl_3_ extract > H_2_O extract > PE extract. Then, the antioxidant ability of five fractions (Fr.1–Fr.5) from EtOAc extract was analyzed, and the result showed the five parts with the IC_50_ values of 3.76 µg/mL, 1.98 µg/mL, 2.46 µg/mL, 3.44 µg/mL, and 4.02 µg/mL, respectively. It was obviously observed that Fr.2 has good antioxidant activity compared to other fractions. In the present study, Fr.2 was selected for further purification, since less is known about the antioxidants in the leaves of this plant.

*TLC-DPPH-ESI/MS of Fr.2*

This active Fr.2 was monitored by TLC-DPPH method to guide the isolation because this method gives quick access for detection and localization of the active compounds in a complicated plant extract. In this study six white yellow spots in the chromatograms were observed on a purple background under visible light (Figure 1B), which have obvious DPPH^. ^scavenging activities. In addition, the same stained TLC plate was also inspected under UV254 (Figure 1A). Note that the antioxidant spots shown in Figure 1A were also observed in those of Figure 1B. It is interesting that a higher free radical scavenging activity was observed in compounds** 2**, **3, **and **5 **compared with other compounds by using the thin-layer chromatography video scanning software to scan the two plats in Figure 2A. Moreover, this paper still analyzed 6 possible activity compounds to speculate their structures by using of ESI/MS technique and comparison of their MS data with the literature data. The structures of 6 possible activity compounds were presented as quercetin-3-*O-α-L*-(3"-galloyl)-rhamnoside **(1)**, quercetin-3-*O-α-L*-(2"-galloyl)-rhamnoside **(2)**,quercetin-3-*O-α-L*-(2"-galloyl)-arabinopyranoside**(3)**, acertannin **(4)**, gallic acid **(5) **and methyl gallate **(6) **in Figure 2B. In 6 compounds **3** of them were not found in AG, such as compound **1**, **2,** and **3** (14, 15). 

*Identification of antioxidant compounds*

Guided isolation through bioautography on TLC using DPPH as a detection reagent led to the isolation of six antioxidant compounds from Fr.2. They were identified as quercetin-3-*O-α-L*-(3"-galloyl)-rhamnoside **(1)**, quercetin-3-*O-α-L*-(2"-galloyl)-rhamnoside **(2)**, quercetin-3-*O-α-L*-(2"-galloyl)-arabinopyranoside**(3)**, acertannin **(4)**, gallic acid **(5)**, and methyl gallate **(6) **by UV, ^1^H, ^13^C-NMR, ESI-MS spectra and by comparison with the literature data (Table.1). The structures of six isolated compounds were in accord with the presumed structures in the analysis of Fr.2 by TLC-DPPH-ESI/MS.

Compound **1** was also obtained as a yellow powder, and gave a positive reaction with HCL-Mg reagent and Molish reaction, probably indicating a flavonoid nature. Its UV spectrum was consistent with that of a flavonoid with maxima at 255, 360 nm. A direct comparison of ^1^H, ^13^C-NMR data (Table1) with the reported data (17) led to identification of **1** as quercetin-3-*O-α-L*-(3"-galloyl)-rhamnoside, which was further confirmed by a positive ESI-MS analysis (m/z 601[M+H]^+^). This is the first report on the isolation of quercetin-3-*O-α-L*-(3"-galloyl)-rhamnoside from genus *Acer*.

Compound **2** was also obtained as a yellow powder, and gave a positive reaction with HCL-Mg reagent and Molish reaction, probably indicating a flavonoid nature. Its UV spectrum was consistent with that of a flavonoid with maxima at 255, 360 nm. A direct comparison of ^1^H, ^13^C-NMR data (Table1) with the reported data (18) led to identification of **2** as quercetin-3-*O-α-L*-(2"-galloyl)-rhamnoside, which was further confirmed by a positive ESI-MS analysis (m/z 601[M+H]^+^). This is the first report on the isolation of quercetin-3-*O-α-L*-(2"-galloyl)-rhamnoside from genus *Acer*.

Compound **3** was also obtained as a yellow powder, and gave a positive reaction with HCL-Mg reagent and Molish reaction, probably indicating a flavonoid nature. Its UV spectrum was consistent with that of a flavonoid with maxima at 255, 360 nm A direct comparison of ^1^H, ^13^C-NMR data (Table1) with the reported data (19) led to identification of 3 as quercetin-3-*O-α-L*-(2"-galloyl)-arabinopyranosid, which was further confirmed by a positive ESI-MS analysis (m/z589[M+H]^+^).This is the first time to report the isolation of quercetin-3-*O-α-L*-(2"-galloyl)-arabinopyranosid from this plant.

Compound **4** was also obtained as a white powder, and gave a positive reaction with FeCl_3 _reagent and Molish reaction, probably indicating a tannins nature. Its UV spectrum was at 275. A direct comparison of ^1^H, ^13^C-NMR data (Table 1) with the reported data (20) led to identification of **4 **as acertannin, which was further confirmed by a positive ESI-MS analysis (m/z 469[M+H]^+^). 

Compound **5** was also obtained as a white powder, and gave a positive reaction with FeCl_3 _reagent. A direct comparison of ^13^C-NMR data (Table 1) with the reported data (21) led to identification of **5** as gallic acid, which was further confirmed by a negative ESI-MS analysis (m/z 171[M+H]^+^). 

Compound **6** was also obtained as a white powder, and gave a positive reaction with FeCl_3 _reagent. A direct comparison of ^13^C-NMR data (Table 1) with the reported data (22) led to identification of **6** as methyl gallate, which was further confirmed by a positive ESI-MS analysis (m/z 185[M+H]^ +^). This is the first time to report the isolation of methyl gallate from this plant.

*DPPH scavenging activity of the isolates*

The antioxidant activities of all the isolated compounds were estimated using the conventional spectrophotometric DPPH^. ^Scavenging capacity assay, 6 compounds were all showed significant DPPH scavenging activities with IC_50_ values of 6.02 μg/mL(1), 2.83 μg/mL **(2)**, 2.34 μg/mL **(3)**, 5.14 μg/mL **(4)**, 1.86 μg/mL **(5)**, and 5.31 μg/mL **(6)**, respectively, which were comparable to that of Vitamin C 1.48μg/mL. The antioxidant activity decreased as follows: Vitamin C>5>3>2>4>6>1. Figure 3 shows the clearance rate curves for the DPPH^. ^scavenging activities of compounds 1-6 and the positive control Vitamin C. Compare to the result reported early only acertannin and methyl gallate were found hand good DPPH scavenging activities. But in this paper 3 another compounds were isolated and showed better antioxidant activity than acertannin and methyl gallate. Especially the DPPH. scavenging activities of compound **5** approached the level of Vitamin C.

## Conclusions

In the present study, a rapid and simple TLC-DPPH-ESI/MS technique has been established that was improved and successfully applied for the investigation of potential antioxidant candidates from the antioxidant parts of *A. ginnala* for the first time. Using this method, the study finally isolated and identified 6 compounds. Compound **1** and **2** were isolated for the first time from genus *Acer*, while compound **3** was isolated for the first time from *A. ginnala*. In addition, the free radical scavenging capacities of the available identified compounds were also investigated and compounds** 2**, **3** and **5** showed significant DPPH^.^Scavenging capacities, with IC_50_ values of 2.83 μg/mL, 2.34 μg/mL, and 1.86 μg/mL, respectively. It is confirmed that this improved approach appeared useful and reliable in rapid screening and identification of natural antioxidants in other biological samples, such as natural products and traditional herbal medicines.
